# The added value of applying a disinvestment approach to the process of health technology assessment in Italy

**DOI:** 10.1017/S0266462323000107

**Published:** 2023-03-02

**Authors:** Chiara Cadeddu, Luca Regazzi, Eugenio Di Brino, Michele Basile, Fidelia Cascini, Andrea Paladini, Filippo Rumi, Americo Cicchetti, Walter Ricciardi

**Affiliations:** 1 Section of Hygiene, Department of Life Sciences and Public Health, Università Cattolica del Sacro Cuore, Rome, Italy; 2 Graduate School of Health Economics and Management (ALTEMS), Università Cattolica del Sacro Cuore, Rome, Italy

**Keywords:** technology assessment, biomedical, health expenditures, health care costs, policy making, Italy

## Abstract

**Objectives:**

The objective of the present policy analysis was to understand how a disinvestment approach to the process of health technology assessment (HTA), applied to the field of medical devices, might help Italian policymakers to properly spend the resources in healthcare.

**Methods:**

Previous international and national experiences in disinvestment for medical devices were reviewed. Precious insights for the rational expenditure of the resources were derived by assessing the evidence available.

**Results:**

The disinvestment of ineffective or inappropriate technologies or interventions with an inadequate value-for-money ratio has become a growing priority for National Health Systems. Different international disinvestment experiences of medical devices were identified and described through a rapid review. Although most of them have a strong theoretical framework, their practical application remains difficult. In Italy, there are no examples of large and complex HTA-based disinvestment practices, but their importance is becoming increasingly acknowledged, especially given the need to prioritize the funds provided by Recovery and Resilience Plan.

**Conclusions:**

Anchoring decisions on health technologies without reassessing the current technological landscape through a robust HTA model might expose to the risk of not ensuring the best employment of the resources available. Thus, it is necessary to develop a strong HTA ecosystem in Italy through adequate consultation with stakeholders to enable a data-driven and evidence-based prioritization of resources toward choices characterized by high value for both patients and society as a whole.

## Introduction

The process of technological innovation in recent years has seen a progressive and unstoppable acceleration, with an incremental impact both on organizational models, as well as costs and expected benefits of National Health Systems (NHS) worldwide, including Italy. The technological and organizational changes that this entails are estimated to have an impact on 50 percent of the total increase in healthcare costs (1–3). As estimated by OECD in 2015, in the next 20 years the increasing trend in health-related costs will reach up to 2 percent of GDP (4), and half of such increase will be linked to the technological innovation process in healthcare. The current economic growth will hardly satisfy such an increase in resources’ consumption; therefore, in order to prevent a reduction in the scope of services provided guarantee the universality and equity of the Italian NHS, alternative strategies must be adopted, including those related to systematic processes designed to appropriately divest obsolete technologies as a fundamental part of an efficient way to prepare the pathway to the introduction of future innovative technologies.

In healthcare, the most consolidated methodology to support the decision-making process aimed to efficiently allocate resources to new technologies (i.e., *medicines, medical devices, assistive technologies, techniques, and procedures developed to solve health problems and improve the quality of life* (5)) is the Health Technology Assessment (HTA) framework. HTA is one of the most recognized tools that allow an appropriate resource allocation, especially through the economic assessment, that brings savings for the healthcare system without affecting the attention to all the relevant issues of a technology (efficacy, organizational aspects, etc.). HTA is applied to macro, meso, and micro (local) levels. This last application is of high practical importance, even if less implemented compared to the other two (6).

The disinvestment of ineffective or inappropriate technologies or interventions with an inadequate value-for-money ratio has become a growing priority for high-quality care and appropriate resource allocation. Indeed, the possibility of reallocating assets from obsolete technologies, allows for an extra source of resources for technological innovation and thus, an increased sustainability of (NHS).

In fact, the ability to reallocate resources from obsolete and no longer appropriate technologies makes it possible to contribute to the recovery of resources to be allocated to technology innovation and thus to the overall sustainability of the system. Effective disinvestment requires a systems approach that, coordinated by a national directorate and implemented at the regional level, must cascade to the organization and delivery of services and benefits by health care providers, which are required to actively involve health care professionals and citizens, particularly in the categories of waste (over- and under-utilization) that are strongly associated with over-medicalization and inadequate transfer of research findings to clinical practice and the organization of health care services. In fact, in the absence of an effective disinvestment process to reduce waste and increase the value of money invested, a substantial share of health spending will continue to produce no return in terms of health, and any additional resources (public or private) will only end up feeding waste.

In Italy, there are no extensive and complex disinvestment experiences employing HTA methodologies while there appear to be several experiences in countries such as the United Kingdom, Canada, USA, Australia, and Spain (7;8).

At the Italian level, the “National HTA Program for medical devices” drafted a strategic document in 2019 to structure a framework in which innovation in the field of medical devices is a standard in the NHS. The guidelines drawn up indicate a well-structured disinvestment process for the assessment of health technologies.

The need to allocate the Recovery and Resilience Plan (RRP) funds in the modernization of the hospital technology equipment for a total investment cost of over €4 billion constitutes an opportunity to implement the national HTA plan by applying the disinvestment process required and shared by all the stakeholders and giving new emphasis to a program that has been inactive for some time. The use of HTA, both to encourage the introduction of innovation and to support disinvestment choices, enables decision makers to make decisions that are rational and reasoned and is supportive in a progressive process of taking responsibilities.

## Objective

The objective of the present policy analysis was to understand how a disinvestment approach to the process of HTA in the field of medical devices might help Italian policymakers in the budgeting and spending prioritization of new resources in healthcare.

## Methods

To reach this aim, we performed a rapid review and a field search to analyze and identify the best practices among the international disinvestment experiences of medical devices described in the literature using the HTA method, both at international and national (Italian) levels. Five scientific databases were consulted, without time limit and for papers published only in English or Italian: (i) PubMed; (ii) Scopus; (iii) EBSCO, including Cochrane Central Register of Controlled Trials, Cochrane Database of Systematic Reviews, HTAs, and NHS Economic Evaluation Database; (iv) CRD Database, including DARE, HTAs, and NHS Economic Evaluation Database; and (v) GIN (Guidelines International Network) database. The records retrieved were analyzed after being subjected to specific exclusion criteria identified previously, which were the topic not related to disinvestment, the absence of relevance related to disinvestment and the unavailability of the full text. The selected evidence was entered into a database created on an Excel worksheet by associating an ID number to each study, as well as the year of publication of the study, the title, the citation, and whether it was a duplicate or not. The studies were then assigned to two blinded researchers (C.C., E.D.B.), who analyzed them by title and abstract. Subsequently, two additional investigators (L.R., A.P.) synthesized the data from the individual selection evidence. Then, we reviewed the priorities outlined by the Italian NextGenerationEU publicly available with regard to the healthcare sector. Finally, we merged these analyses to derive precious insights for the implementation of NextGenerationEU.

## Results

### International Experiences in Disinvestment

Issues related to the disinvestment approach are relatively recent and started to be addressed in the late 2000s, which explains the lack of internationally standardized procedures. The first article about disinvestment in healthcare was published in Australia in 2007 (9). The same year, the Victoria’s Government published a discussion paper about the future directions for health technology uptake, diffusion, and disinvestment (10). Since then, other organizations such as the Swedish Council on Technology Assessment in Health Care (SBU), the West Scotland NHS, and the Canadian Agency for Drugs and Technologies in Health (CADTH), have shown interest in this area.

The problem of a lack of guidance on how to conduct the overall process of disinvestment was described in 2010 by Ibargoyen-Roteta et al. (7) reporting results about the development of the GuNFT guideline and highlighting the need for disinvestment to be a guided process. This work can be considered as a first guideline designing a scenario to disinvest in established health technologies. In 2018, Calabrò et al. (11) agreed that the disinvestment process posed challenges from a methodological point of view, and that to tackle these challenges, it was necessary to standardize methodologies and procedures at an international level, developing new methodological approaches to produce and grow evidence on disinvestment policies and practices. In 2019, Esandi et al. (12) noted that the disinvestment process poses an unprecedented challenge for the HTA movement, as it requires an iterative reassessment in the recourse to any technology once adopted and implies the need for developing and implementing new strategies and methodologies to face this demand. In particular, Esandi et al. identified three main challenges for the HTA agencies worldwide:

- the enormous number of separate interventions currently available that might be selected for disinvestment;
- the overload due to the continuously growing number of new technologies to assess, which limits the possibility to monitor existing items;
- the lack of a uniform process to select and prioritize potential candidates for disinvestment.

On the basis of their evidence, to address these issues, Esandi et al. proposed a new framework to provide support to HTA organizations and governments working in the field of disinvestment, proposing seven basic approaches (i.e., the ways organizations deal with the multiplicity and diversity of existing technologies whose *value-for-money* is not well established or uncertain), a set of triggers (i.e., criteria that, when present, allow the identification of potential candidates for disinvestment) and two types of methods (i.e., the methodological strategies an organization considers for applying triggers or identification criteria).

Over the last two decades, other studies were conducted internationally to develop a homogenous framework for disinvestment.

Two studies by Esmail et al. (13) and by Soril et al. (14) described the health technology reassessment (HTR) as an important process that, along with Knowledge Translation (KT), can be used to optimize technology use throughout its lifecycle, including the disinvestment phase. HTR is defined as *a structured, evidence-based assessment of the clinical, social, ethical, and economic effects of a technology, currently used in the healthcare system, to inform optimal use of that technology in comparison to alternatives* and is intended as a mechanism to improve patient care and system efficiency by reallocating resources from low-value care to higher value interventions and technologies. HTR should be guided by the dynamic process of KT, which can fill the gap between *what we should be doing* and *what we are actually doing* and should imply two fundamental elements: the concrete engagement of stakeholders, and the discussion and application of updated knowledge, both fundamental for a fruitful HTR application.

Seo et al. (15) reviewed the status of HTR carried out in seven countries (Spain, Denmark, Sweden, United Kingdom, Canada, United States of America, and Australia) and found various similarities in the HTR processes:

- presence of HTA agencies;
- identification and priority setting of the candidate technology for reassessment;
- stakeholder engagement;
- support related to coverage reimbursement;
- strategies for implementation.

They subsequently developed a practical HTR framework for the South Korean healthcare system composed of four steps: identification, prioritization, reassessment, and decision.

The *Guideline for Not Funding existing health Technologies in healthcare systems* (GuNFT) (16) developed by the Basque Office for HTA (Osteba) is the only transparent, systematic, and explicit procedure to evaluate the likelihood of disinvestment for some health technologies. According to these guidelines, the funding of a technology is not recommended if overall general deterioration in patients’ health is demonstrated, if the potential risks related to the technology overcome benefits or if it causes relevant discomfort or a negative impact on patients’ health. The GuNFT guideline process for disinvestment consists of seven phases:

- identification;
- validation of applications;
- prioritization (if necessary);
- assessment of applications;
- decision making;
- development of an action plan;
- diffusion of the decision, the reasons why it has been taken and the action plan to achieve disinvestment.

However, despite being the only currently available guideline for disinvestment, data about its implementation are scarce and mainly related to facility-based or hospital services, or not available online (17).

Another possible approach for disinvestment is the Program Budgeting and Marginal Analysis (PBMA), used for the priority setting in healthcare since 1970s in several countries with different health systems (18). The first step of the PBMA consists in examining the current expenditure of resources (i.e., program budgeting) and then focus on marginal health gains and costs of modifications in that expenditure (i.e., marginal analysis), by means of comparisons within or across programs. After this step, the contents produced are analyzed by a multi-disciplinary panel of managers, clinicians, and other stakeholders to define a list of options to change the current pattern of resource allocation. Thanks to the *marginal analysis*, it is possible to shift the resources accessible to the healthcare organizations to more useful programs for the achievement of organizations’ strategic goals (19).

Finally, one last international example of approach to disinvestment in healthcare is the Australian SHARE (Sustainability in Health care by Allocating Resources Effectively) program (20). Its goal was to create a complex system and process for the disinvestment decision-making and resource allocation, characterized by an organization-wide, systematic, integrated, transparent, and evidence-based approach, for the application to a local healthcare setting (Monash Health).

The SHARE Program starts from the SEAchange model for Sustainable, Effective, and Appropriate change in health services, which implies four phases:

- the identification of the need for change;
- the development of a proposal to address the need;
- the implementation of the proposal;
- the evaluation of the degree and influence of the change.

For each phase, the evidence-based practice values are stressed to guarantee that the process is carried out by considering the best available evidence from studies and applied data, the experience and expertise of healthcare professionals and the values and viewpoints of consumers. Moreover, it is always considered the sustainability, avoidance of duplication, and integration of new modalities into current systems. An action research component allows a constant exploration of the change course to advance the current project and inform the work for the future.

Other relevant experiences to be cited can be found from Latin America. In particular, they can be mainly referred to the guidelines of the Brazilian Ministry of Health relating to the disinvestment process (22), also cited by Guerra-Júnior et al. (23), and to the study by Agirrezabal et al. (24).

The guidelines of the Brazilian Ministry of Health relating to the disinvestment process illustrate the Health Technology Performance Assessment (HTpA) method. The indication of analysis for HTpA may arise from the health system management, from society or academia. It is interesting how the disinvestment process is carried out and agreed upon during the incorporation. The disinvestment procedures must automatically modify and update the guidelines. The disinvestment process should also be preceded by a moment of public reflection, preferably using the method of public consultation (22).

In relation to these guidelines, the study by Guerra-Júnior et al. highlights the need of creating evidence for the introduction of new health technologies which are often approved on the basis of the results of Randomized Controlled Trials, which are controlled settings. The importance of using a methodology that could assess and measure the impact of a technology in a real-world setting, in the specific health system where the technology is used, is thus highly relevant and the HTpA method is a possible example of this application (23).

The study by Agirrezabal et al. is a collection of all the best practices applied for the disinvestment of health technologies in Latin America. The work has been carried out through a systematic review of the literature supported by an expert opinion survey. The results show some local and focused experiences and underline the lack of literature on the topic of disinvestment together with a low representation of experts describing practices of disinvestment of health technologies in their working setting (24).

The results above described are summarized in Table 1.

**Table 1.** Summary of most relevant international experiences in disinvestment identified through our rapid review

References	Model	HTA methodology	Top-down or bottom-up approach	Main results
Soril et al. (14)	HTR – Knowledge translation	Yes	Top-down and bottom-up	In the paper by Soril et al. (14), there is a practical framework, composed of shared topics systematized in six main domains about: value of the existing technology; present employment gap; current instruments and resources; drivers of change; expected results; foundational stakeholders. These six domains described by specific questions allow to help resolving the HTR issues for the advance of its understanding, as well as to its implementation and identification of the process to advance in the modern healthcare system
Goodwin et al. (19)	Programme Budgeting and Marginal Analysis (PBMA)	No	Bottom-up	The study by Goodwin et al. (19) clearly provided strategic and operational priorities by means of the application of PBMA for 2010/11. It addressed the staff with focus and structure, obtaining also a relevant planned reduction in hospital activity levels. Participants demonstrated to be satisfied about the procedure. The Plymouth National Health Service followed the PBMA process, even if opened discussions related to the evidence for some priorities, decibel rationing, and missing robust challenge at meetings for priority-setting. The authors stated that to improve participants’ understanding of marginal analysis, further work is needed. Participants underlined many external benefits, especially for cultural change, and recognized that the process should involve the full local health and social care community. This estimation specifies that the methodology for priority-setting was effective for the selection of priorities for NHS Plymouth and that PBMA represents a proper process for the local allocation of resources. However, for an effective application aimed at identifying savings, it is necessary to remove cultural and structural barriers to disinvestment
Harris et al. (20)	SHARE	No	Top-down	The SHARE programme consists of two phases and four steps. The first phase includes the first step of the identification of the need for change and the second about the development of proposal for change. In the second phase the two final steps deal with the implementation of change proposal and the evaluation of the outcomes of change. This framework provides values for decision-making and settings that give chances to introduce systematic prompts and triggers to start disinvestment practices to a local level
Ibargoyen-Roteta et al. (7)	GuNFT guideline	Yes	Bottom-up	In the GuNFT guideline, six domains are described: (i) general initial recommendations, (ii) completing the application form, (iii) examination and priority-setting of the applications, (iv) assessment, (v) final decision, and (vi) action plan design. A software is also available for enabling an easier and guided GuNFT application for the disinvestment process
Brazilian Ministry of Health (22)	Health technology performance assessment (HTpA)	Yes	Bottom-up	HTpA refers to the systematic evaluation of the properties, effects, and/or impact of a health intervention or health technology in the real world to provide information for investment/disinvestment decisions and clinical guideline updates. HTpA may indicate the need to disinvest in one or more of the four modalities: (i) Full delisting, (ii) Restriction, (iii) Retraction, and (iv) Substitution

HTA, health technology assessment; HTR, health technology reassessment.

### Italian Experiences in Disinvestment

In the Italian context, there are no prior examples of large and complex disinvestment practices using HTA methodologies. However, it is likely that in the development of disinvestment strategies particular attention should be paid to the identification of an integrated model between the different levels of the NHS (national, regional, and local). For this reason, the implementation of effective disinvestment strategies in the future will probably require a systematic approach coordinated by a national body that can be implemented at a regional level and can be extended to an organizational level, including health organizations, hospitals, and other service providers.

In Italy, some first steps toward the definition of a national framework for disinvestment were undertaken by the Italian Society for Health Technology Assessment (SIHTA) and by the Italian Group for Evidence-Based Medicine (GIMBE) Foundation.

In 2013, SIHTA has openly dealt with the theme of disinvestment in its annual Health Policy Forum, introducing the HTA as a tool for priority setting and disinvestment, as well as an alternative to the concept of a spending review, which had become a reality for the Italian NHS following the economic and political crisis of 2011. Specifically, SIHTA organized three working groups on three different themes regarding the methods for disinvestment, the role of stakeholders, and the role of HTA. The knowledge and expertise produced by these working groups were then employed to produce a steering document for the National HTA Program Control Room (*Cabina di Regia del Programma Nazionale di HTA*) (21), which was established by combining the contributions of the Italian Ministry for Health, the National Agencies for Regional Health Services and for Medicines (AGENAS and AIFA) and the Regions to coordinate the HTA activities developed at national and local level, implementing a National Program. In fact, the Pact for Health 2014–2016 and the Stability Laws of 2015 and 2016 outlined a new institutional model of cooperation between central and regional levels for the definition of behaviors aimed at achieving objectives of clinical efficacy, management efficiency, and sustainability of innovation (25).

The Control Room proposed to further develop and articulate the *reporting system* in order to identify the *cost-saving* technologies through a bottom-up or top-down approach. In the first case, the technologies that can potentially be divested are reported by the stakeholders (scientific societies/patient associations/healthcare companies, etc.), while in the top-down approach, the reporting is carried out by dedicated units that identify the technologies eligible for disinvestment (i.e., horizon scanning and a reassessment process)

Previously, in 2016, on its 11th National Conference, the GIMBE Foundation presented the *Framework GIMBE for disinvestment in healthcare* (26), subject to a memorandum of understanding signed with the Italian National Agency for Regional Health Services (AGENAS) (27).

To address the problem of overuse and under-use, the GIMBE framework aims to divest from ineffective, inappropriate, and low-value health services and reallocate the recovered resources into effective, appropriate, and under-utilized high-value services, through tools and actions acting on the following determinants: health (re-)planning, knowledge translation, information and active involvement of citizens and patients. Considering the peculiarities of the Italian NHS, with its multiple levels of governance (national, regional, local, and health provider), the GIMBE framework distinguishes disinvestment actions that can be taken at various levels:

- at the national and regional level, disinvestment for some health interventions can be conditioned by regulatory interventions (exclusion from the Essentials Levels of Assistance, revocation of accreditation, exclusion from the formulary, sustainability thresholds for high-cost drugs, etc.), expression of the implementation of the allocative dimension of value through tools such as HTA and value-based pricing;
- at the local and healthcare provider level, the disinvestment process is conditioned both by the health interventions offered and by the doctor–patient relationship, so that effective disinvestment from low-value technologies, procedures, and care paths (defined through an appropriate HTA process) can be obtained only implementing strategies for changing professional behaviors and promote the tools of shared decision-making to increase the personal dimension of value.

**Figure 1.**
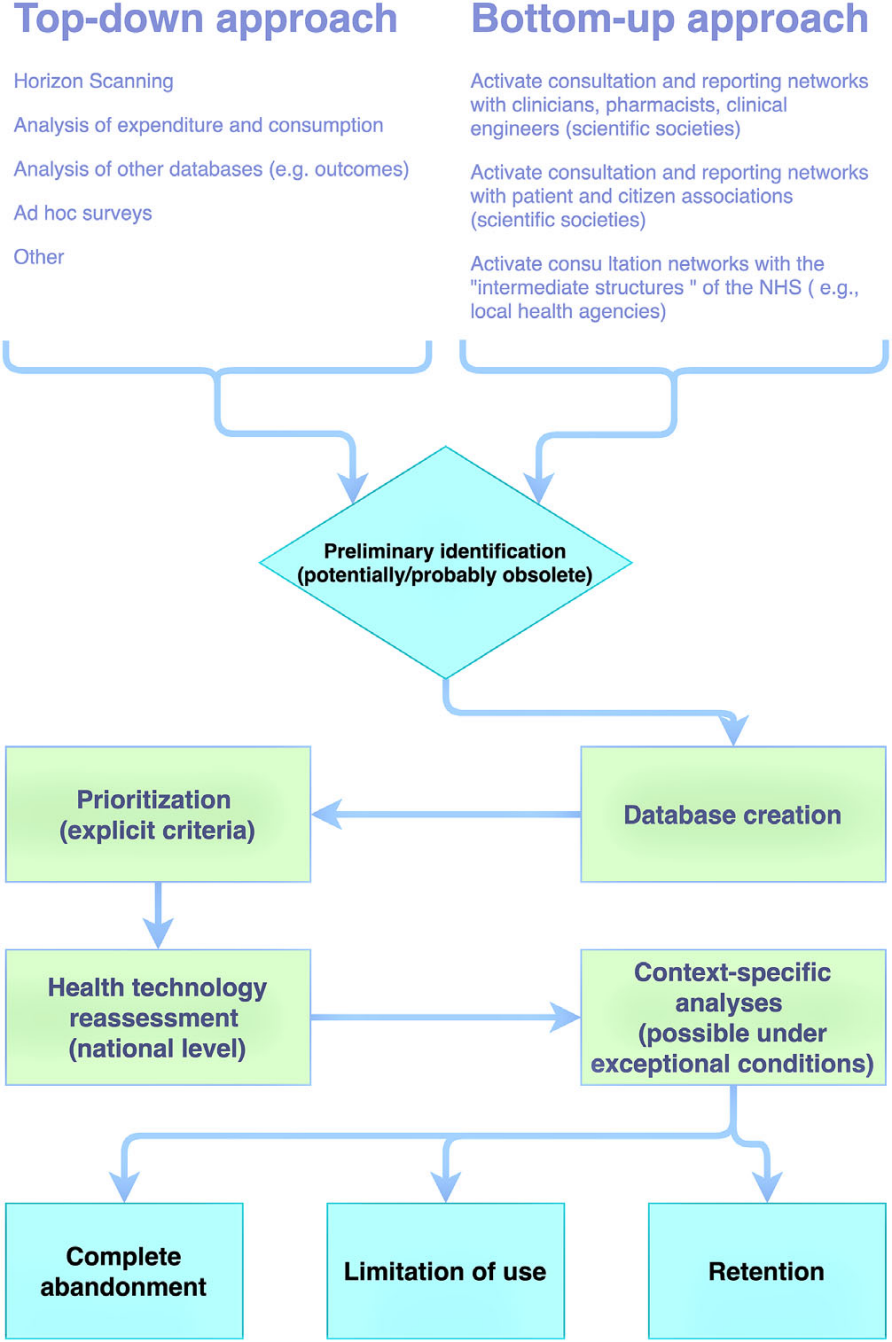
Disinvestment process scheme for medical devices in Italy. Adapted from Ministero della Salute (28).

The disinvestment process within the national HTA program of medical devices in Italy is summarized in Figure 1. Consistently with the HTA framework, stakeholder involvement is also required for disinvestment.

It involves the following six stages:

- identification of candidate technologies for divestment and reporting (identification can take place through a top-down or a bottom-up approach, allowing for the collection of preliminary information to be verified together with the producers of the technologies).
- prioritization of candidate technologies.
- re-evaluation of prioritized technologies.
- final decision and communication.
- implementation through the creation or adaptation of international guidelines to the Italian contest is envisaged to strengthen the final decision taken and communicated in the previous phase.
- implementation monitoring.

In both proposed models (top-down and bottom-up), technology reporting is done through a tab preset consisting of four sections detailing: the stakeholder requesting the initiation of the disinvestment process assessment, technical characteristics of the technology under consideration and its current use, and the macro and micro reasons for the disinvestment request.

### The Future of HTA and Disinvestment in Italy

The adoption of a disinvestment approach appears to be particularly urgent in the Italian context, especially in light of the new funds made available by Next Generation EU (29).

As a matter of fact, a large part of the technological landscape currently in use was introduced without a HTA logic or with heterogeneous HTA logics among regions, so most of the technologies already available would benefit from a reassessment for it is at greater risk of inappropriateness, lack of convenience, and neglect of optimal useful life.

The realization of an appropriate, sustainable, and shared investment plan would require a multidisciplinary and multi-stakeholder assessment inspired by a robust HTA process.

The current limitation lies in the fact that the disinvestment methodologies described in this paper have never reached the operational phase in Italy. The Italian divestment model described in the “National HTA program for medical devices” could finally find a useful practical application in the modernization of the hospital technology equipment envisaged in component 2 of Mission 6 “Health” of the RRP.

Two recently approved implementing decrees of the Italian Government (DL.vo August 5, 2022, No. 137 and 138) seem to go in this direction, as they aim to ensure compliance with the provisions dictated by Regulations (EU) 2017/745 and 2017/746 (concerning the functioning of the internal EU market of medical devices) by all involved parties (notified bodies, economic operators and healthcare professionals) and include an explicit reference to the relaunch of the National HTA Program of Medical Devices and the application of disinvestment.

## Conclusions

Disinvestment processes have the purpose of optimizing the allocation of resources by eliminating practices considered of little value, replacing them with procedures of an adequate value-for-money ratio. In the absence of an effective disinvestment process to reduce waste, increase efficiency, and produce a higher return on the expenditures realized, a substantial share of health expenditure will continue to produce low or no return in terms of health, and any additional resources (public or private) may only end up supporting obsolete technologies. Being the output of the process of evaluating or re-evaluating technologies, disinvestment is to be considered as an approach consistent with the need to keep the technological park qualitatively updated on the frontier of effectiveness, efficiency and characterized by the lowest possible clinical and organizational risk.

Besides of the existence of standardized methodologies in the field of disinvestment in countries like Spain, Australia, Canada, Denmark, South Korea, Sweden, and the United Kingdom, further research is necessary to establish and/or organize a consistent ground for disinvestment on a wider scale, specifically for the use of disinvestment processes in a coordinated manner at a national scale. This is especially important for Italy, given the great amount of financial resources that the RRP invested in the renovation of the health technology park. Thus, it is pivotal that the National HTA Program of Medical Devices is recovered and that a standardized framework for disinvestment is applied in the decision process to allocate the RRP resources.
